# Thrombocytopenia and platelet course on hospital mortality in neurological intensive care unit: a retrospective observational study from large database

**DOI:** 10.1186/s12883-020-01794-1

**Published:** 2020-05-30

**Authors:** Dawei Zhou, Zhimin Li, Lei Wu, Guangzhi Shi, Jianxin Zhou

**Affiliations:** Department of Critical Care Medicine, Beijing Tiantan Hospital, Capital Medical University, Beijing, China

**Keywords:** Neurological ICU, Thrombocytopenia, Platelet count, Mortality

## Abstract

**Background:**

Thrombocytopenia (TP) has been shown to be an independent predictor of mortality in the intensive care unit (ICU) patients. Studies are lacking in the neurological ICU (NICU) population. The aim was to evaluate the incidence of TP in NICU and the relationship between TP and outcomes.

**Methods:**

We conducted a retrospective multicenter study of prospectively collected data of all patients admitted to the NICU between 2014 and 2015 from a large database (eICU Collaborative Research Database). The main exposure was TP at admission and TP developed during NICU stay. Multivariable logistic regression and Cox proportional hazard models were used to evaluate the relationship of TP at admission and platelet course with hospital mortality. The primary outcome was hospital mortality.

**Results:**

7450 patients in NICU from 17 hospitals were included. Hospital mortality was 9%. TP at admission was present in 20% of patients, TP developed during NICU stay was present in 13.2% of patients. TP at admission was not associated with hospital mortality after adjusting for confounders (OR 1.14 [95% CI 0.92–1.41, *p* = 0.237]). Hospital mortality of continuous TP during NICU stay was 15% while hospital mortality of recovery from TP at admission was 6% (*p* < 0.001). Patients with TP developed during NICU stay had higher odds ratio for hospital mortality (OR 1.65 [95% CI 1.3–2.09, *p* < 0.001]).

**Conclusions:**

Thrombocytopenia is common in NICU and patients who have thrombocytopenia not resolving have increased mortality. Patients’ recovery from thrombocytopenia may predict a good prognosis.

## Background

Thrombocytopenia (TP) is one of the most common problems in different kinds of intensive care units (ICUs), such as medical ICU, surgical ICU, medical/surgical ICD, pediatric ICU, neonatal ICU, coronary ICU, cardiac surgical ICU, and has been considered to play an important role in worsening the prognosis of ICU patients [1, 2]. TP incidence varies from different types of ICU and across different groups of the patient populations, ranged from 13.3 to 66.7% [1, 3]. However, no such study has been carried out in the neurological ICU (NICU).

TP has been found associated with increased bleeding episodes and increased transfusion requirements of platelets, fresh frozen plasma, and red blood cells and has also been associated with prolonged ICU and hospital stays and increased mortality in critically ill patients [4–9]. Neurological diseases with TP may be associated with poor outcomes [10–13]. The relationship between TP and outcomes in neurological critically ill patients is still unknown.

To determine the prevalence and significance of TP in NICU, we studied patients admitted to multicenter NICUs. The main objectives of the present study were to investigate the incidence of TP in NICU and the relationship of TP at admission and platelet course with hospital mortality in patients admitted in NICU.

## Methods

### Setting

This study used data stored in the eICU (eicu-crd.mit.edu) database with 200,859 admissions between 2014 and 2015 at 208 hospitals located across the United States. The elaborate description of eICU is available elsewhere [14]. The schema of eICU was established in collaboration with Privacert (Cambridge, MA), who certified the re-identification risk as meeting safe harbor standards (Health Insurance Portability and Accountability Act [HIPPA] Certification no. 1031219–2). All tables in eICU were deidentified to meet the safe harbor provision of the US HIPAA. Due to the HIPAA compliant de-identification in this database, our institutional review board (IRB) requirement was waived. After completing a National Institutes of Health (NIH) web-based training course (Protecting Human Research Participants), the author (certification number: 28795067) was approved to access to the database for research aims.

### Study population

All patients in the eICU were eligible for inclusion in the present investigation. As for those who admitted to ICU for more than once, only the first ICU stay was taken into consideration. We selected all adult patients admitted to NICU. Patients were excluded for the following reasons: (1) Missing hospital mortality data, (2) Missing platelet count data during the first 24 h, (3) Platelet measurement only once during NICU stay.

### Clinical variables and outcomes

Data on the following information were extracted: demographics (age, gender, body mass index [BMI] and, ethnicity), comorbidities (hypertension, diabetes mellitus, heart failure, respiratory disease, cirrhosis, chronic renal insufficiency, and cancer), physiological parameters (including vital signs and laboratory tests) over the first 24 h in the NICU, platelet count during the whole NICU stay, Acute Physiology and Chronic Health Evaluation (APACHE) IV score, Glasgow Coma Scale (GCS), life support interventions (e.g., use of mechanical ventilation, vasopressors), treatments (red blood cells transfusion, platelet transfusion, mannitol, hypertonic saline, heparin, and glucocorticoid), NICU mortality, locations at hospital discharge (dead, home, nursing home, skilled nursing facility, rehabilitation, and others), and duration of hospital and ICU stay.

Platelet count data during the whole NICU stay were extracted. Thrombocytopenia (TP) was defined as a platelet count below 150 ×  10^9^/L and nonthrombocytopenia (NTP) was a platelet count ≥150 × 10^9^/L. According to TP at admission (the first platelet count value during the first 24 h) and platelet course (i.e. TP developed during the rest of NICU stay), four groups were defined. They were Group 1 (TP-TP), Group 2 (TP-NTP), Group 3 (NTP-TP), and Group 4 (NTP-NTP). Group 1 (TP-TP): TP at admission and during the rest of NICU stay; Group 2 (TP-NTP): TP at admission and at least one episode of NTP during the rest of NICU stay; Group 3 (NTP-TP): NTP at admission and at least one episode of TP during the rest of NICU stay; Group 4 (NTP-NTP): NTP at admission and during the rest of NICU stay.

The main outcome measure was hospital mortality, defined as the status of patient survival at the time of hospital discharge. The second outcomes were NICU mortality, NICU length of stay and hospital length of stay.

### Statistical analysis

Data were initially assessed for normality. Continuous variables were shown as mean and standard deviation (SD) or median and interquartile range (IQR). Categorical variables were reported as numbers and percentages. Groups of different platelet count were compared by the chi-square test for categorical variables and Kruskal-Wallis test or analysis of variance for continuous ones. Survivors and non-survivors data were compared using t-tests or Mann-Whitney U tests for continuous variables and chi-square tests or Fisher’s exact test for categorical variables. Variables with equal to or greater than 40% missing values were excluded from the analysis (Supplemental figure 1). Multiple imputation was performed for the remaining variables with missing data [15].

To investigate the independent effect of TP at admission and platelet course on hospital mortality, multivariable regression models were used using logistic regression for hospital death and Cox proportional hazards regression for time to death. Hospital mortality was considered as a time-to-event variable. The event was death during hospitalization. A patient was censored when discharged alive. Two models were created: model 1 adjusted for APACHE IV score; model 2 adjusted for APACHE IV score, admission diagnosis, whether the patient had heart failure, hypertension, cancer, chronic renal insufficiency, cirrhosis, use of mechanical ventilation, vasopressors, mannitol, hypertonic saline, heparin, glucocorticoid, red blood cell transfusion, and platelet transfusion. These covariates were selected as a priori because they were potential confounders as determined by subject-matter knowledge. Results were presented as odds ratio (ORs) with confidence intervals (CIs) for logistic regression and hazard ratios (HRs) with CIs for Cox proportional hazards regression. Duration of survival was presented as Kaplan-Meier curves with log-rank tests comparing the equality of groups.

Data extraction was performed using PostgreSQL (version 10, www.postgresql.org). R software (version 3.5.1, www.r-project.org) was used for statistical analysis. A two-sided *P* value of < 0.05 was considered statistically significant.

## Results

**Fig. 1:**
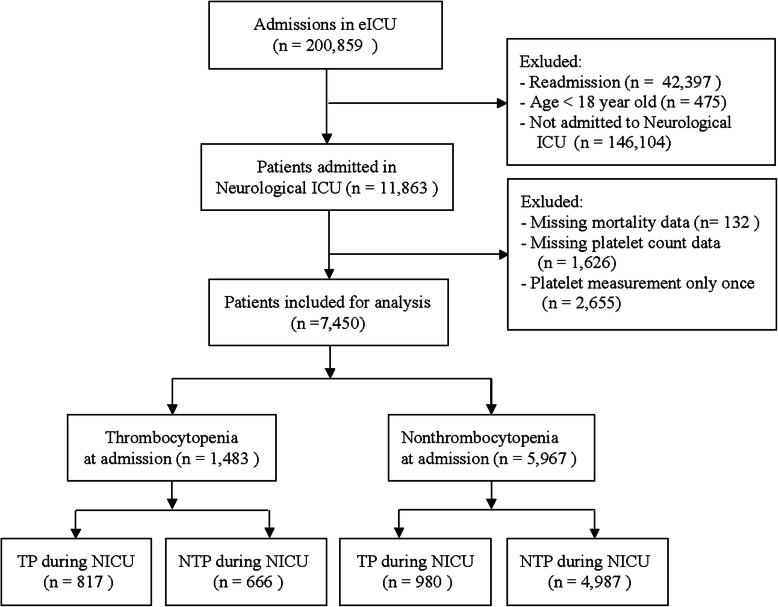
Flow chart of subject selection. ICU intensive care unit, TP thrombocytopenia, NTP nonthrombocytopenia. TP-TP: TP at admission and during the rest of NICU stay; TP-NTP: TP at admission and at least one episode of NTP during the rest of NICU stay; NTP-TP: NTP at admission and at least one episode of TP during the rest of NICU stay; NTP-NTP: NTP at admission and during the rest of NICU stay

The eICU database contained 200,839 patient admissions. After exclusion, a total of 7450 patients from 17 hospitals were analyzed in our study (Fig. 1), including 6804 survivors and 646 non-survivors, giving hospital mortality 9%. NICU mortality was 4%. The main disease categories in the study were postoperation (23%), ischemic stroke (17%), intracranial hemorrhage (10%), traumatic brain injury (8%). Baseline characteristics of study patients between survivors and non-survivors were presented in Supplemental Table 1. Platelet count during the first 14 days between survivors and non-survivors was presented in Supplemental figure 3. As expected, non-survivors had significantly greater severity of illness as represented by APACHE IV and GCS scores. Non-survivors had lower platelet count during the first 12 days.

TP at admission was present in 1483 (20%) patients, of which 666 patients had at least one episode of NTP during the rest of NICU stay. TP developed during the rest of NICU stay was present in 980 (13.2%) patients. Overall, 2463 (33.2%) patients had at least one episode of TP during the whole NICU stay. Comparisons of different groups of platelet count were displayed in Table 1. Group 1 (TP-TP) had older age, lower BMI, a higher percentage of heart failure, cirrhosis, chronic renal insufficiency, and cancer. Group 4 (NTP-NTP) had the lowest APACHE IV score and the highest GCS score. The four groups had statistically different percentage of male sex.

**Table 1:** Baseline characteristics during the first 24 h in the NICU

	TP – TP (*N* = 817)	TP – NTP (*N* = 666)	NTP – TP (*N* = 980)	NTP – NTP (*N* = 4, 987)	*P* value
Age, years (median, [IQR])	67 (56, 78)	65 (52, 76)	64 (51, 75)	63 (50, 75)	< 0.001
Gender: male (n (%))	537 (66)	401 (60)	544 (56)	2467 (49)	< 0.001
BMI (median, [IQR])	26.6 (23.2, 31.4)	27.4 (23.4, 32)	27.4 (23.6, 32.3)	27.5 (23.5, 32.4)	0.019
Ethnicity (n (%))					0.504
Caucasian	621 (76)	498 (75)	720 (73)	3669 (74)	
African American	108 (13)	98 (15)	143 (15)	731 (15)	
Hispanic	32 (4)	21 (3)	41 (4)	192 (4)	
Asian	15 (2)	12 (2)	15 (2)	74 (1)	
Native American	10 (1)	2 (0)	4 (0)	38 (1)	
Other/Unknown	31 (4)	35 (5)	57 (6)	283 (6)	
Comorbidities (n (%))					
Hypertension	401 (49)	322 (48)	484 (49)	2422 (49)	0.96
Diabetes mellitus	190 (23)	130 (20)	222 (23)	1131 (23)	0.288
Respiratory disease	87 (11)	65 (10)	74 (8)	465 (9)	0.139
Heart failure	105 (13)	76 (11)	90 (9)	342 (7)	< 0.001
Cirrhosis	82 (10)	18 (3)	17 (2)	31 (1)	< 0.001
Chronic renal insufficiency	116 (14)	66 (10)	96 (10)	295 (6)	< 0.001
Cancer	208 (25)	103 (15)	145 (15)	633 (13)	< 0.001
Disease category (n (%))					< 0.001
Postoperation	162 (20)	169 (25)	234 (24)	1128 (23)	
Ischemic stroke	97 (12)	82 (12)	121 (12)	964 (19)	
Traumatic brain injury	75 (9)	62 (9)	94 (10)	341 (7)	
Intracranial hemorrhage	65 (8)	51 (8)	97 (10)	551 (11)	
Hematoma subdural	54 (7)	32 (5)	42 (4)	234 (5)	
Epilepsy	42 (5)	35 (5)	41 (4)	216 (4)	
Multiple Trauma	37 (5)	38 (6)	59 (6)	137 (3)	
Subarachnoid hemorrhage	15 (2)	12 (2)	26 (3)	185 (4)	
Neoplasm	19 (2)	6 (1)	26 (3)	215 (4)	
Others	251 (31)	179 (27)	240 (24)	1016 (20)	
Vital signs, (median, [IQR])					
Maximum Temperature, °C	37.2 (36.9, 37.7)	37.3 (37, 37.9)	37.4 (37, 37.9)	37.2 (36.9, 37.6)	< 0.001
Minimum Temperature, °C	36.4 (36.1, 36.6)	36.5 (36.1, 36.7)	36.4 (36.1, 36.7)	36.5 (36.2, 36.7)	< 0.001
Maximum HR, beats/min	98 (86, 113)	103 (89, 119)	104 (90, 120)	99 (87, 112)	< 0.001
Minimum HR, beats/min	66 (58, 77)	67 (59, 79)	68 (59, 79)	65 (58, 75)	< 0.001
Maximum RR	26 (23, 30)	26 (23, 31)	26 (23, 31)	26 (23, 30)	0.034
Minimum RR	12 (10, 14)	12 (10, 14)	12 (10, 14)	12 (10, 14)	0.076
Maximum MBP, mmHg	104 (95, 117)	105 (95, 119)	106 (96, 120)	107 (97, 121)	< 0.001
Minimum MBP, mmHg	64 (55, 73)	64 (56, 73)	64 (56, 72)	67 (59, 76)	< 0.001
Laboratory test, (median, [IQR])					
Maximum hemoglobin, g/dl	12.2 (10.5, 13.8)	12.5 (11.1, 14)	13 (11.3, 14.5)	12.9 (11.4, 14.2)	< 0.001
Minimum hemoglobin, g/dl	10.84 (8.5, 12.5)	10.9 (9.1, 12.5)	11.4 (9.5, 13.1)	11.9 (10.3, 13.2)	< 0.001
Maximum WBC, × 10^9^/L	8.9 (6.3, 13.1)	10.8 (7.9,14.3)	12.9 (9.7, 16.8)	11.89 (9, 15.4)	< 0.001
Minimum WBC, × 10^9^/L	6.6 (4.7, 9.3)	8 (6, 10.1)	9.5 (7.1, 12.7)	9.4 (7.4, 12.2)	< 0.001
Disease severity, (median, [IQR])					
APACHE IV score	53 (41, 71)	52 (39, 68)	55 (38, 72)	44 (33, 59)	< 0.001
GCS	14 (10, 15)	14 (9, 15)	13 (8, 15)	14 (11, 15)	< 0.001

Data are median (interquartile range) or No / Total (%)

NICU Neurological intensive care unit, *TP* Thrombocytopenia, *NTP* Non thrombocytopenia, *IQR* Interquartile range, *BMI* Body mass index, *HR* Heart rate, *RR* Respiratory rate, *MAP* Mean arterial pressure, *WBC* White blood cell, *APACHE* Acute Physiology and Chronic Health Evaluation, *GCS* Glasgow coma scale

Table 2 showed treatments and outcomes by the platelet count category. Group 1 (TP-TP) and Group 3 (NTP-TP) had higher hospital mortality and NICU mortality than Group 2 (TP-NTP) and Group 4 (NTP-NTP). Group 3 (NTP-TP) had the highest proportion of use of mechanical ventilation (38%) and vasopressors (15%). Group 1 (TP-TP) had the highest RBC (6%) and platelet (4%) transfusions, and the lowest proportion of use of hypertonic saline (1%), mannitol (1%) and heparin (4%). Group 2 (TP-NTP) had the highest proportion of the use of heparin (10%).

**Table 2:** Treatments and outcomes by platelet count category

	TP – TP (*N* = 817)	TP – NTP (*N* = 666)	NTP – TP (*N* = 980)	NTP – NTP (*N* = 4, 987)	*P* value
Treatments, (n (%))					
Mechanical Ventilation	186 (23)	232 (35)	373 (38)	1037 (21)	< 0.001
Use of vasopressors	76 (9)	67 (10)	149 (15)	254 (5)	< 0.001
RBC transfusion	45 (6)	37 (6)	41 (4)	65 (1)	< 0.001
Platelet transfusion	31 (4)	14 (2)	12 (1)	43 (1)	< 0.001
Hypertonic saline	8 (1)	16 (2)	43 (4)	96 (2)	< 0.001
Mannitol	9 (1)	10 (2)	40 (4)	93 (2)	< 0.001
Glucocorticoid	40 (5)	26 (4)	56 (6)	298 (6)	0.128
Heparin	31 (4)	68 (10)	82 (8)	379 (8)	< 0.001
Outcomes					
ICU mortality, n (%)	61 (7)	23 (3)	87 (9)	156 (3)	< 0.001
Hospital mortality, n (%)	125 (15)	42 (6)	158 (16)	321 (6)	< 0.001
ICU length of stay, d, median, [IQR]	2 (2, 4)	4 (2, 8)	4 (3, 9)	3 (2, 5)	< 0.001
Hospital length of stay, d, median, [IQR]	6 (4, 9)	10 (7, 16)	9 (6, 16)	6 (4, 10)	< 0.001
Hospital discharge location among survivors, n (%)					< 0.001
Home	395 (48)	239 (36)	363 (37)	2546 (51)	
Rehabilitation	53 (6)	105 (16)	123 (13)	613 (12)	
Nursing Home	4 (0)	7 (1)	7 (1)	24 (0)	
Skilled Nursing Facility	140 (17)	146 (22)	167 (17)	809 (16)	
Other	100 (12)	127 (19)	162 (17)	674 (14)	

*TP* Thrombocytopenia, *NTP* Non thrombocytopenia, *IQR* Interquartile range, *RBC* Red blood cell, *ICU* Intensive care unit

Table 3 showed the ORs and HRs of hospital mortality in crude and adjusted models. TP at admission was associated with hospital mortality in both logistic and Cox proportional hazards regression models without adjusting for covariates. Supplemental figure 2 displayed the Kaplan-Meier survival curves by TP at admission, which showed that TP at admission was associated with a lower probability of survival (log-rank *p* = 0.002). But after adjusting for covariates in model 1 and 2, TP at admission was not associated with hospital mortality.

**Table 3:** Crude and adjusted odds or hazard ratio for hospital mortality (logistic regression and Cox-proportional hazards) in patients with different platelet count categories

Categories	Logistic Regression Analysis		Cox Proportional Hazards Model	
	OR (95% CI)	*P* value	HR (95% CI)	*P* value
Platelet count at admission				
Crude				
NTP at admission	1 [Reference]		1 [Reference]	
TP at admission	1.45 (1.2–1.75)	0.002	1.32 (1.11–1.57)	0.002
Model 1				
NTP at admission	1 [Reference]		1 [Reference]	
TP at admission	1.02 (0.83–1.26)	0.819	1.03 (0.86–1.24)	0.715
Model 2				
NTP at admission	1 [Reference]		1 [Reference]	
TP at admission	1.14 (0.92–1.41)	0.237	1.15 (0.95–1.38)	0.143
Platelet course during NICU stay				
Crude				
Group 4 (NTP-NTP)	1 [Reference]		1 [Reference]	
Group 1 (TP-TP)	2.63 (2.1–3.27)	< 0.001	1.27 (1.11–1.44)	< 0.001
Group 2 (TP-NTP)	0.98 (0.78–1.25)	0.897	1.03 (0.89–1.19)	0.711
Group 3 (NTP-TP)	2.79 (2.27–3.42)	< 0.001	1.32 (1.15–1.53)	< 0.001
Model 1				
Group 4 (NTP-NTP)	1 [Reference]		1 [Reference]	
Group 1 (TP-TP)	1.73 (1.35–2.21)	< 0.001	1.97 (1.59–1.44)	< 0.001
Group 2 (TP-NTP)	0.63 (0.44–0.89)	0.011	0.47 (0.34–0.66)	< 0.001
Group 3 (NTP-TP)	1.82 (1.45–2.27)	< 0.001	1.22 (1.00–1.48)	0.052
Model 2				
Group 4 (NTP-NTP)	1 [Reference]		1 [Reference]	
Group 1 (TP-TP)	2.05 (1.57–2.66)	< 0.001	2.37 (1.89–2.98)	< 0.001
Group 2 (TP-NTP)	0.65 (0.48–0.93)	0.021	0.49 (0.35–0.68)	< 0.001
Group 3 (NTP-TP)	1.65 (1.30–2.09)	< 0.001	1.08 (0.88–1.33)	0.438

Model 1used Acute Physiology and Chronic Health Evaluation IV score as covariate

Model 2 used Acute Physiology and Chronic Health Evaluation IV score, admission diagnosis, whether the patient had heart failure, hypertension, cancer, chronic renal insufficiency, cirrhosis, use of mechanical ventilation, vasopressors, mannitol, hypertonic saline, heparin, glucocorticoid, red blood cell transfusion and platelet transfusion as covariates

*OR* Odd ratio, *CI* Confidential interval, *HR* Hazard ratio, *TP* Thrombocytopenia, *NTP* Non thrombocytopenia, *NICU* Neurological intensive care unit

**Fig. 2:**
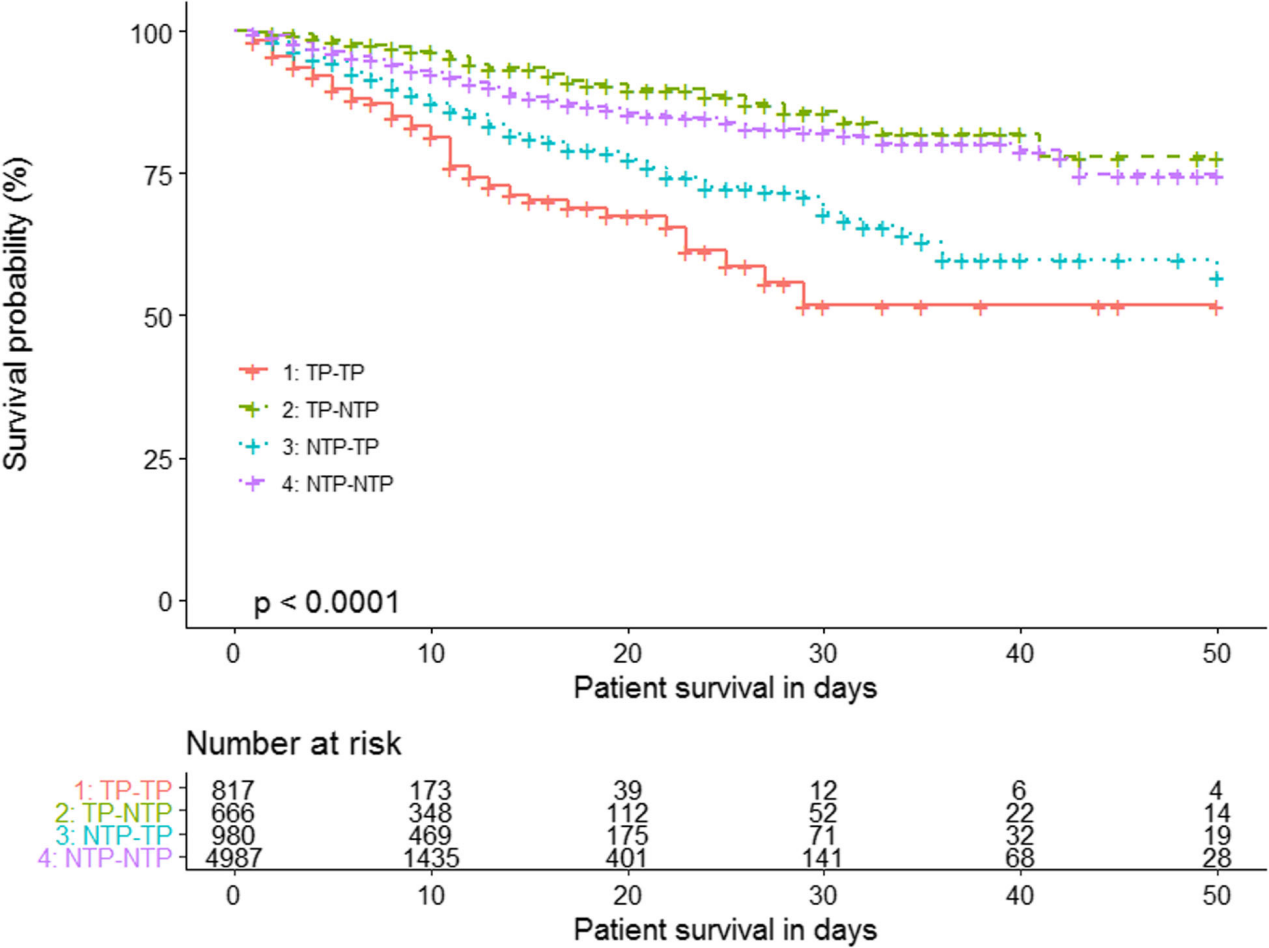
Kaplan-Meier survival curves by platelet count at admission and platelet course category. TP thrombocytopenia, NTP nonthrombocytopenia. TP-TP: TP at admission and during the rest of NICU stay; TP-NTP: TP at admission and at least one episode of NTP during the rest of NICU stay; NTP-TP: NTP at admission and at least one episode of TP during the rest of NICU stay; NTP-NTP: NTP at admission and during the rest of NICU stay

Compared with Group 4 (NTP-NTP), Group 1 (TP-TP) patients were associated with higher hospital mortality both in crude and adjusted models; Group 2 (TP-NTP) patients were not associated with hospital mortality in crude models, but after adjusting for covariates, were associated with lower hospital mortality both in logistic and Cox proportional hazards regression models; Group 3 (NTP-TP) patients were associated with higher hospital mortality in crude models, and after adjusting for covariates, were associated with higher hospital mortality in logistic regression model but had no statistically significant difference in Cox proportional hazards regression model (Table 3). Figure 2 displayed the Kaplan-Meier survival curves of different groups, which showed that Group 2 (TP-NTP) was associated with the highest probability of survival and Group 1 (TP-TP) was associated with the lowest probability of survival (log-rank *p* < 0.001).

## Discussion

The main findings were summarized as follows. TP was a common problem in NICU. TP at admission was present in 20% of patients, developed during NICU stay was present in 13.2% of patients, thus 33.2% of patients had at least one episode of TP during the whole NICU stay. TP at admission was not associated with hospital mortality after adjusting for confounders. Continuous TP during the whole NICU stay was associated with higher hospital mortality. Patients’ recovery from TP at admission during NICU stay may predict a good prognosis. Patients with new onset TP during NICU stay had a higher odds ratio for hospital mortality.

TP is one of the most common hematological abnormalities encountered in ICU, both at admission or during the course of ICU stay [2, 16]. Incidence varies from different ICUs with medical ICU ranged from 36 to 67% [4, 5], surgical ICU from 13 to 41% [17, 18], surgical/ medical ICU from 25 to 46% [19–21], coronary/ cardiac Surgery ICU from 19 to 21% [22, 23], neonatal/ pediatric ICU from 22 to 25% [24–26]. The threshold for defining TP is inconsistent, with some studies defining it as a platelet count < 100,000/ μL, and this may be one of the reasons for wide variation. In the present study, TP at admission was present in 20% of patients admitted to NICU, 13.2% of patients developed TP during the course of the NICU stay, and 33.2% of the patients had at least one episode of TP.

The reasons for TP in ICU are numerous and often multifactorial mechanisms act simultaneously. The mechanisms contributing to TP in the ICU include the following: (1) pseudothrombocytopenia, (2) hemodilution, (3) platelet consumption, (4) decreased production, (5) increased sequestration of platelet, and (6) immune-mediated destruction of platelet [9]. Sepsis and trauma are the most common causes of TP in the ICU [16]. For ICU patients with TP, resulted from a disturbance of the balance among platelet production, pooling, and consumption, often more than one of these mechanisms is responsible for the low platelet count [16]. In NICU, the main diseases were postoperation, stroke, traumatic brain injury, intracranial hemorrhage, epilepsy and so on [27]. There may be some different mechanisms of TP from other kinds of ICUs. For example, valproic acid is known to cause both immune and non-immune-mediated TP [28].

TP is associated with an increased risk of mortality in critically ill patients [4–6, 19, 20, 29–31]. Platelet count changes in the critically ill may have a biphasic pattern that is significantly different in patients with good and those with poor prognosis [20, 32]. Some studies showed non-resolution of TP, but not TP itself was associated with mortality [17, 33]. In the present study, TP at admission was not associated with hospital mortality. Although NICU patients who had continuous TP during NICU stay had the more severe disease at admission, the association between continuous TP and hospital mortality remained highly significant after adjusting for APACHE IV score and other covariates. In multivariable logistic regression models after adjusting confounders, patients with new onset TP during NICU stay had a higher odds ratio for hospital mortality. Thus, TP-TP and NTP-TP appears to be a predictor of mortality in NICU, independent of the severity of disease at admission. The relationship of TP and platelet course with hospital mortality is unlikely to be causal. More likely, the disease causes and contributes to TP. However, some severity scoring systems such as APACHE IV do not take platelet count into account [34].

TP was associated with a poor prognosis of many neurological diseases. The main consequence of TP is the perceived risk of bleeding. Patients with TP were more likely to develop major bleeding episodes and they also received more blood product transfusions [33]. A nadir platelet count below 50 × 10^9^/L was identified as an independent risk factor for bleeding [35]. TP is a risk factor for the progression of hemorrhagic injuries in TBI [13]. In cancer patients with cerebrovascular disease, TP at the time of cerebrovascular disease diagnosis was associated with higher mortality [10].

In the Kaplan-Meier survival curves of different platelet count groups, group 2 (TP-NTP) was associated with the highest probability of survival. After adjusting for confounders, group 2 (TP-NTP) had a lower odds ratio and hazard ratio for hospital mortality. That was interesting. In the study of Venkata et al., they also found patients with TP resolved by hospital discharge had lower ICU, hospital and 28-day mortality than those who did not resolve [33]. Akca et al. found a relative increase in platelet count after TP was present in survivors but not in non-survivors [20]. Nijsten et al. found a blunted or absent rise in platelet count in critically ill patients was associated with increased mortality [17]. These results showed changes in platelet count over time may have good prognostic significance.

The strength of the present study is the large sample size that allows for the adjustment of multiple confounding factors. Secondly, the subjects of the study came from multicenter NICUs, making the results applicable to heterogeneous NICU patients. However, there are several obvious limitations to this study. First, the study was retrospective in nature and subject to the inherent limitations of the design. This study design only allowed us to show statistical associations and not causality between platelet count and hospital mortality. Second, although we used multivariable logistic and Cox proportional hazard regression models to adjust for potential confounders, many potential confounding factors might not have been included in the analysis, leading to biased results. Third, in eICU, all patients admitted from the Operating room or Recovery room had a postoperative diagnosis, even though some surgical patients have a medical reason for admission. That may to some extent change the disease spectrum. Fourth, the mortality rate was relatively low in the present cohort [36]. Patients with neurological disease may be admitted in other ICUs and vice versa, making our results less generalizable. Fifth, we excluded 2655 patients with only one platelet count value, and the reasons for having only platelet count value are unknown. Among potential factors, reasons could have been due to premature death or rapid recovery. In our study, the reason may be inclined to rapid recovery (Supplemental Table 2). Finally, the database only had data between 2014 and 2015 right now. Treatment and diagnosis may change over time, which may influence the platelet course.

## Conclusions

In this multicenter observational study, TP is common in the NICU. TP at admission was present in 20% of patients, and 33.2% had at least one episode of TP during the whole NICU stay. Patients with TP at admission not resolving during NICU stay have increased mortality, while recovery to NTP may predict a good prognosis. Patients with new onset TP during NICU had a higher odds ratio for hospital mortality.

## Supplementary information

**Additional file 1: Table S1.**
Characteristics of study patients between survivors and non-survivors. **Table S2.** Characteristics of patients between included and excluded by number of platelet count. **Figure S1.** Missing rate for variables extracted from the database. **Figure S2.** Kaplan-Meier survival curves by platelet count at admission category. TP thrombocytopenia, NTP nonthrombocytopenia. **Figure S3.** Daily platelet count in the survivors and non-survivors.

## Data Availability

Data analyzed during the present study are currently stored in the eICU database (eicu-crd.mit.edu). After completing the required training course (the Collaborative Institutional Training Initiative) and requesting access to the eICU Collaborative Research Database, researchers can seek to use the database.

## Abbreviations

APACHE: Acute Physiology and Chronic Health Evaluation
BMI: Body mass index
GCS: Glasgow Coma Scale
HIPPA: Health Insurance Portability and Accountability Act
HR: Hazard ratio
ICU: Intensive care unit
IQR: Interquartile range
IRB: Institutional review board
MV: Mechanically ventilated
NICU: Neurological intensive care unit
NIH: National Institutes of Health
NTP: Nonthrombocytopenia
OR: Odds ratio
SD: Standard deviation
TP: Thrombocytopenia
