# Supplementary material for: Thrombocytopenia and platelet course on hospital mortality in neurological intensive care unit: a retrospective observational study from large database
Source: BMC Neurol. 2020 May 30;20:220. doi: 10.1186/s12883-020-01794-1 (PMC7260747; doi:10.1186/s12883-020-01794-1)
Supplement: Supplementary file 1 — Additional file 1: Table S1. Characteristics of study patients between survivors and non-survivors. Table S2. Characteristics of patients between included and excluded by number of platelet count. Figure S1. Missing rate for variables extracted from the database. Figure S2. Kaplan-Meier survival curves by platelet count at admission category. TP thrombocytopenia, NTP nonthrombocytopenia. Figure S3. Daily platelet count in the survivors and non-survivors. [file 12883_2020_1794_MOESM1_ESM.docx]

**Thrombocytopenia and platelet course on hospital mortality in neurological intensive care unit: a retrospective observational study from large database**

**Supplemental Materials**

Dawei Zhou^1^, Zhimin Li^1^, Lei Wu^1^, Guangzhi Shi^1^, Jianxin Zhou^1^

1. Institution: Department of Critical Care Medicine, Beijing Tiantan Hospital, Capital Medical University, Beijing, China.

Supplemental table 1 Characteristics of study patients between survivors and non-survivors

| Variables | Total  (n = 7, 450) | Survivors  (n =6, 804) | Non-Survivors  (n = 646) | P value |
| --- | --- | --- | --- | --- |
| Age, years (median, [IQR]) | 64 (51, 75) | 63 (50, 75) | 69 (57, 79) | <0.001 |
| Gender: male (n (%)) | 3949 (53) | 3599 (53) | 350 (54) | 0.474 |
| BMI (median, [IQR]) | 27.4 (23.5, 32.3) | 27.5 (23.5, 32.3) | 26.5 (22.9, 31.8) | 0.002 |
| Ethnicity (n (%)) |  |  |  | 0.34 |
| Caucasian | 5508 (74) | 5047 (74) | 461 (72) |  |
| African American | 1080 (14) | 987 (14) | 93 (14) |  |
| Hispanic | 286 (4) | 261 (4) | 25 (4) |  |
| Asian | 116 (2) | 102 (1) | 14 (2) |  |
| Native American | 54 (1) | 49 (1) | 5 (1) |  |
| Other/Unknown | 406 (5) | 361 (5) | 45 (7) |  |
| Comorbidities (n (%)) |  |  |  |  |
| Hypertension | 3629 (49) | 3291 (48) | 338 (52) | 0.06 |
| Diabetes mellitus | 1673 (22) | 1517 (22) | 156 (24) | 0.272 |
| Respiratory disease | 691 (9) | 620 (9) | 71 (11) | 0.122 |
| Heart failure | 613 (8) | 543 (8) | 70 (11) | 0.013 |
| Cirrhosis | 148 (2) | 126 (2) | 22 (3) | 0.01 |
| Chronic renal insufficiency | 573 (8) | 501 (7) | 72 (11) | 0.001 |
| Cancer | 1089 (15) | 969 (14) | 120 (19) | 0.003 |
| Disease category (n (%)) |  |  |  | <0.001 |
| Postoperation | 1693 (23) | 1613 (24) | 80 (12) |  |
| Ischemic stroke | 1264 (17) | 1156 (17) | 108 (17) |  |
| Traumatic brain injury | 572 (8) | 504 (7) | 68 (11) |  |
| Intracranial hemorrhage | 764 (10) | 658 (10) | 106 (16) |  |
| Hematoma subdural | 362 (5) | 333 (5) | 29 (5) |  |
| Epilepsy | 334 (4) | 321 (5) | 13 (2) |  |
| Multiple Trauma | 271 (4) | 261 (4) | 10 (2) |  |
| Subarachnoid hemorrhage | 238 (3) | 211 (3) | 27 (4) |  |
| Neoplasm | 266 (4) | 257 (4) | 9 (1) |  |
| Others | 1686 (23) | 1493 (22) | 193 (30) |  |
| Vital signs, (median, [IQR]) |  |  |  |  |
| Maximum Temperature, °C | 37.2 (36.9, 37.7) | 37.2 (36.9, 37.7) | 37.6 (37.1, 38.2) | <0.001 |
| Minimum Temperature, °C | 36.4 (36.2, 36.7) | 36.5 (36.2, 36.7) | 36.3 (35.8, 36.7) | <0.001 |
| Maximum HR, beats/min | 100 (88, 114) | 99 (87, 113) | 111 (96.5, 126) | <0.001 |
| Minimum HR, beats/min | 66 (58, 76) | 66 (58, 76) | 70 (60, 80) | <0.001 |
| Maximum RR | 26 (23, 30) | 26 (23, 30) | 28 (24, 33) | <0.001 |
| Minimum RR | 12 (10, 14) | 12 (10, 14) | 12 (10, 15) | <0.001 |
| Maximum MBP, mmHg | 107 (97, 120) | 106 (97, 120) | 109 (97, 122) | 0.033 |
| Minimum MBP, mmHg | 66 (58, 75) | 67 (58, 75) | 62 (52, 71) | <0.001 |
| Laboratory test, (median, [IQR]) |  |  |  |  |
| Maximum hemoglobin, g/dl | 12.8 (11.2, 14.2) | 12.8 (11.3, 14.2) | 12.6 (10.7, 14.1) | 0.002 |
| Minimum hemoglobin, g/dl | 11.7 (9.9, 13.1) | 11.7 (10, 13.1) | 10.9 (8.9, 12.5) | <0.001 |
| Maximum WBC, × 10^9^/L | 11.61 (8.6, 15.4) | 11.48 (8.5, 15) | 13.7 (10.3, 18.5) | <0.001 |
| Minimum WBC, × 10^9^/L | 9 (6.8, 11.83) | 8.9 (6.8, 11.7) | 9.9 (7.37, 13.6) | <0.001 |
| Disease severity, (median, [IQR]) |  |  |  |  |
| APACHE IV score | 47 (34, 63) | 45 (34, 60) | 75 (58, 95) | <0.001 |
| GCS | 14 (10, 15) | 14 (11, 15) | 8 (4, 14) | <0.001 |
| Treatments, (n (%)) |  |  |  |  |
| Mechanical Ventilation | 1828 (25) | 1445 (21) | 383 (60) | <0.001 |
| Use of vasopressors | 546 (7) | 393 (6) | 153 (24) | <0.001 |
| RBC transfusion | 188 (3) | 166 (2) | 22 (3) | 0.165 |
| Platelet transfusion | 100 (1) | 81 (1) | 19 (3) | <0.001 |
| Hypertonic saline | 163 (2) | 128 (2) | 35 (5) | <0.001 |
| Mannitol | 152 (2) | 104 (2) | 48 (7) | <0.001 |
| Heparin | 560 (8) | 509 (7) | 51 (8) | 0.762 |
| Glucocorticoid | 420 (6) | 391 (6) | 29 (5) | 0.227 |

Data are median (interquartile range) or No / Total (%).

IQR interquartile range, BMI body mass index, HR heart rate, RR respiratory rate, MAP mean arterial pressure, WBC white blood cell, APACHE Acute Physiology and Chronic Health Evaluation, GCS Glasgow coma scale, RBC red blood cell.

Supplemental table 2 Characteristics of patients between included and excluded by number of

platelet count

| Variables | Total  (n =10,105) | Included  (n =7, 450) | Excluded  (n = 2, 655) | P value |
| --- | --- | --- | --- | --- |
| Age, years (median, [IQR]) | 63 (50, 75) | 64 (51, 75) | 61 (47, 72) | <0.001 |
| Gender: male (n (%)) | 5273 (52) | 3949 (53) | 1324 (50) | 0.006 |
| BMI (median, [IQR]) | 27.4 (22.9, 33.2) | 27.4 (22.5, 33.7) | 27.4 (23.8, 32.1) | 0.245 |
| Ethnicity (n (%)) |  |  |  | <0.001 |
| Caucasian | 7599 (75) | 5508 (74) | 2091 (79) |  |
| African American | 1335 (13) | 1080 (14) | 255 (10) |  |
| Hispanic | 353 (3) | 286 (4) | 67 (3) |  |
| Asian | 167 (2) | 116 (2) | 51 (2) |  |
| Native American | 83 (1) | 54 (1) | 29 (1) |  |
| Other/Unknown | 568 (6) | 406 (5) | 162 (6) |  |
| Disease category (n (%)) |  |  |  | <0.001 |
| Postoperation | 2251 (22) | 1693 (23) | 558 (21) |  |
| Ischemic stroke | 1688 (17) | 1264 (17) | 424 (16) |  |
| Traumatic brain injury | 804 (8) | 572 (8) | 232 (9) |  |
| Intracranial hemorrhage | 945 (9) | 764 (10) | 181 (7) |  |
| Hematoma subdural | 496 (5) | 362 (5) | 134 (5) |  |
| Seizure | 437 (4) | 334 (4) | 103 (4) |  |
| Multiple Trauma | 342 (3) | 271 (4) | 71 (3) |  |
| Subarachnoid hemorrhage | 391 (4) | 238 (3) | 153 (6) |  |
| Neoplasm | 613 (6) | 266 (4) | 347 (13) |  |
| Others | 2138 (21) | 1686 (23) | 452 (17) |  |
| Outcomes |  |  |  |  |
| ICU mortality, n (%) | 420 (4) | 327 (4) | 93 (4) | 0.056 |
| Hospital mortality, n (%) | 812 (8) | 646 (9) | 166 (6) | <0.001 |
| ICU length of stay, d,  median, [IQR] | 2 (2, 4) | 3 (2, 5) | 2 (1, 2) | <0.001 |
| Hospital length of stay, d, median, [IQR] | 6 (3, 10) | 7 (4, 11) | 3 (2, 4) | <0.001 |
| Hospital discharge location among survivors, n (%) |  |  |  | <0.001 |
| Home | 5430 (54) | 3543 (48) | 1887 (71) |  |
| Rehabilitation | 1018 (10) | 894 (12) | 124 (5) |  |
| Nursing Home | 47 (0) | 42 (1) | 5 (0) |  |
| Skilled Nursing Facility | 1507 (15) | 1262 (17) | 245 (9) |  |
| Other | 1291 (13) | 1063 (14) | 228 (8) |  |

IQR interquartile range, BMI body mass index, ICU intensive care unit.


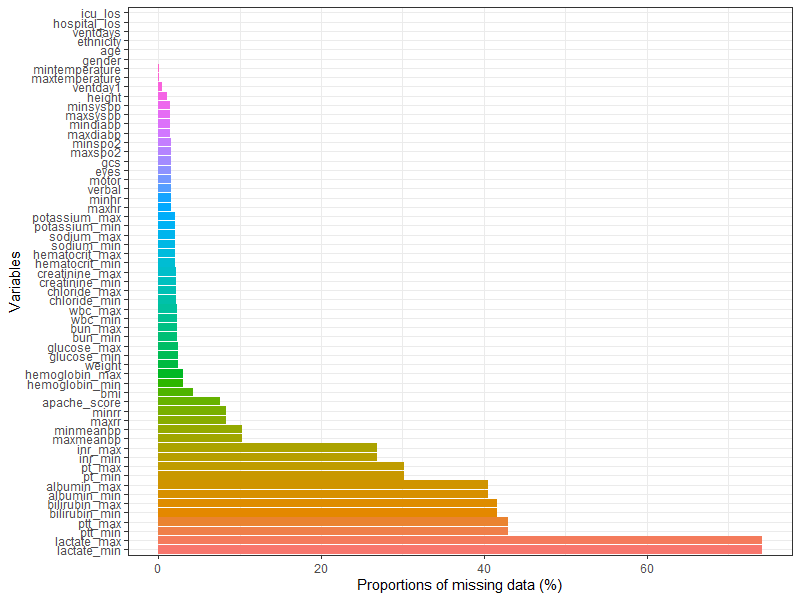


Supplemental figure 1 Missing rate for variables extracted from the database.


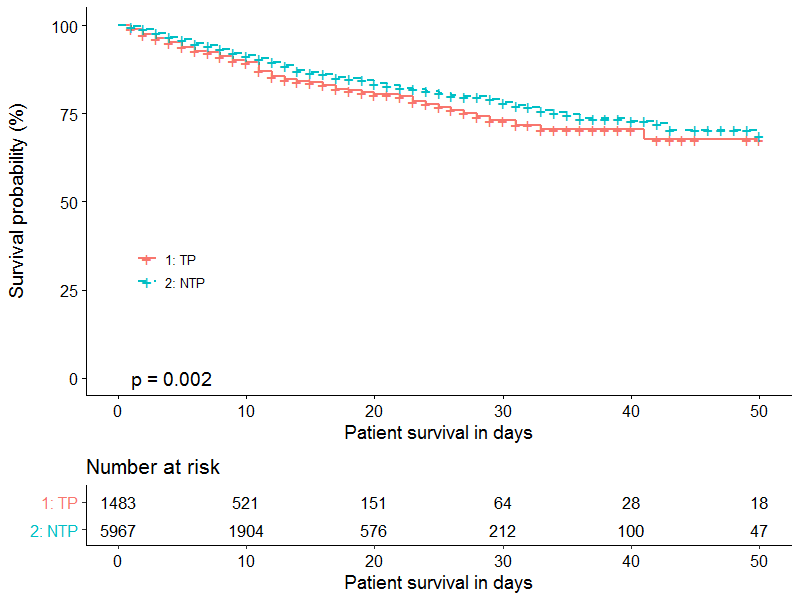


Supplemental figure 2 Kaplan-Meier survival curves by platelet count at admission category. TP thrombocytopenia, NTP nonthrombocytopenia.


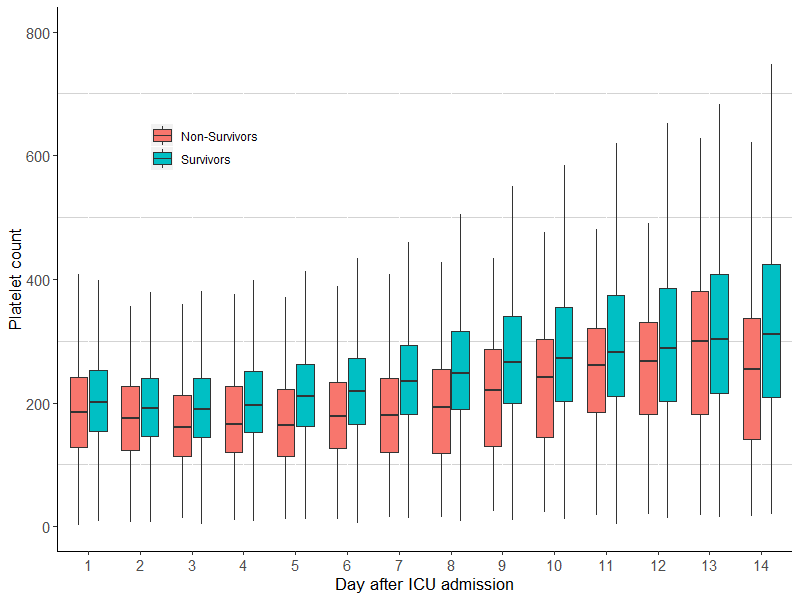


Supplemental figure 3 Daily platelet count in the survivors and non-survivors.
